# Comparative epidemiology of six respiratory pathogens among children aged 28 days to 14 years with acute respiratory infections in coastal Xiamen and inland Linxia, China: a multicenter retrospective study

**DOI:** 10.3389/fpubh.2026.1926943

**Published:** 2026-09-03

**Authors:** Xingxiang Zheng, Hongmei Ma, Jiali Cao, Jumei Liu, Rui Guo, Qunshan Xu, Xiaoli Chen, Shiqiang Han, Huiming Ye

**Affiliations:** 1 Department of Laboratory Medicine, Fujian Key Clinical Specialty of Laboratory Medicine, Women and Children’s Hospital, School of Medicine, Xiamen University, Xiamen, China; 2 State Key Laboratory of Vaccines for Infectious Diseases, Xiang An Biomedicine Laboratory, School of Life Sciences, School of Public Health, Xiamen University, Xiamen, China; 3 Linxia Hui Autonomous Prefecture Maternal and Child Health Hospital, Linxia, China

**Keywords:** acute respiratory infection, child, China, comparative epidemiology, respiratory pathogens

## Abstract

**Objective:**

Acute respiratory infections are a major public health problem in children. The epidemiology of pediatric respiratory pathogens has shifted in recent years. This study aimed to compare the epidemiological patterns of six common respiratory pathogens among children with acute respiratory infections (ARI) in Xiamen and Linxia, China.

**Methods:**

This retrospective study included 5,835 children with ARI from December 2024 to November 2025. Oropharyngeal swabs were tested for rhinovirus (RV), respiratory syncytial virus (RSV), influenza A virus (Flu A), influenza B virus (Flu B), adenovirus (ADV), and *Mycoplasma pneumoniae* (MP) using multiplex RT-PCR. Detection rates were compared by region, sex, age group and season.

**Results:**

Overall, 3,143 samples (53.9%) were positive; 290 (5.0%) had co-detections. RV (23.8%), RSV (20.2%) and Flu A (8.8%) were most frequently detected. Positivity was higher in Linxia than in Xiamen (65.4% vs. 49.0%; *p* < 0.001). After adjustment, Linxia remained associated with higher odds of detection (aOR 1.86, 95% CI 1.65–2.10). Detection was also associated with male sex, toddler/preschool age and autumn/winter seasons.

**Conclusion:**

RV, RSV and Flu A were the most frequently detected pathogens in both regions. The findings indicate regional, age-related and seasonal heterogeneity, supporting locally tailored surveillance and prevention planning.

## Introduction

Acute respiratory infections (ARIs) remain a major public health problem and a leading cause of morbidity and mortality in children worldwide, particularly among children younger than 5 years of age (1–4). Respiratory viruses and atypical bacterial pathogens are among the most common causes of pediatric ARIs (5–8). Common pathogens include influenza A and B viruses, respiratory syncytial virus (RSV), adenovirus (ADV), rhinovirus (RV), and *Mycoplasma pneumoniae* (MP), all of which contribute substantially to the burden of respiratory illness in children. Transmission patterns are influenced by pathogen-specific characteristics as well as host, environmental, climatic, and socioeconomic factors (9).

China includes diverse climatic and geographic settings that may influence the circulation of respiratory pathogens. Xiamen is a coastal city in southeastern China with a subtropical maritime monsoon climate, relatively high humidity and mild seasonal variation. Linxia Hui Autonomous Prefecture is an inland plateau region in northwestern China with cold temperate semi-arid climate or cold temperate semi-humid climate, marked seasonal variation and cold winters. The two settings also differ substantially in altitude, population density, urbanization, socioeconomic profile and healthcare resources. These contrasts provide an opportunity to examine whether contemporaneous pathogen detection patterns differ between coastal and inland pediatric populations, while recognizing that a regional comparison cannot isolate the effect of geography from other contextual factors.

Most epidemiological studies of pediatric respiratory pathogens in China have focused on single regions or similar climate zones, and direct comparisons between coastal and inland regions remain limited (10). This question is especially relevant after the relaxation of COVID-19 non-pharmaceutical interventions, when the circulation patterns of common respiratory pathogens have shifted (11). However, the extent to which post-pandemic detection patterns differ across contrasting Chinese settings remains incompletely described.

We hypothesized that the prevalence and seasonal distribution of detected respiratory pathogens would differ between the two settings. This retrospective multicenter study compared the detection patterns of six common respiratory pathogens among children with ARIs in Xiamen and Linxia from December 2024 to November 2025 and assessed whether the regional difference persisted after adjustment for sex, age group and season. The six-target panel was selected because these pathogens are frequently detected and clinically relevant in pediatric ARIs and were routinely tested at both participating hospitals. The objective was to provide evidence for regionally tailored surveillance and prevention planning, rather than to attribute the observed differences to geography alone.

## Materials and methods

### Study setting, design and population

Xiamen is a densely populated, highly urbanized coastal city, whereas Linxia is a less densely populated inland plateau prefecture with a substantial rural population and a mean elevation of approximately 2,000 m. In 2025, Xiamen and Linxia had permanent populations of 5.365 million and 2.072 million, urbanization rates of 91.31 and 43.09%, and per-capita disposable incomes of CNY 77,762 and CNY 18,566, respectively. Additional population, socioeconomic and healthcare-resource indicators are summarized in Supplementary Table S1 (12, 13). These aggregate indicators describe the administrative regions rather than individual participants or hospital catchments and were not entered into the regression models.

This retrospective study used a convenience sample of respiratory specimens from 5,835 children with clinician-diagnosed ARIs who attended Xiamen Maternal and Child Health Hospital in Fujian Province or Maternal and Child Health Hospital of Linxia Hui Autonomous Prefecture in Gansu Province between December 1, 2024 and November 30, 2025. This was a retrospective analysis, no *a priori* sample-size calculation was performed; all eligible tested children during the prespecified period were included. The study population included 3,458 boys (59.3%) and 2,377 girls (40.7%), with ages ranging from 28 days to 14 years. Sex was extracted from medical records and analyzed as male or female.

Children were eligible if they were aged 28 days to 14 years, met standard pediatric clinical criteria for ARI based on relevant clinical guidance (14), underwent respiratory pathogen testing during the visit and had complete clinical and laboratory data. Children were excluded if the visit or hospitalization was attributable to non-infectious conditions, such as airway foreign body, acute asthma exacerbation or congenital airway malformation, or if hospital-acquired infection was confirmed.

Age was categorized as infants (<1 year, *n* = 2,306), toddlers (1 to <3 years, *n* = 1,655), preschool children (3 to <6 years, *n* = 1,278) and school-aged children (6–14 years, *n* = 596). Seasons were classified according to month of onset as spring (March–May, *n* = 1,383), summer (June–August, *n* = 1,214), autumn (September–November, *n* = 1,705) and winter (December–February of the following year, *n* = 1,533).

### Specimen collection and pathogen detection

Oropharyngeal swab specimens were collected using sterile disposable flocked swabs from the tonsillar pillars or posterior pharyngeal wall. Swabs were placed immediately into tubes containing preservation medium for testing.

Multiplex reverse transcription polymerase chain reaction (RT-PCR) was used to detect six common respiratory pathogens: RV, RSV, Flu A, Flu B, ADV and *Mycoplasma pneumoniae* (MP). The assay targeted conserved regions of each pathogen, enabling simultaneous detection of multiple pathogens in a single reaction.

### Definitions

The overall detection rate was calculated as the number of samples positive for at least one pathogen divided by the total number of tested samples. Pathogen-specific detection rates were calculated as the number of samples positive for each pathogen divided by the total number of tested samples. Because more than one pathogen could be detected in a single specimen, pathogen-specific rates may sum to more than the overall positivity rate. Co-detection was defined as detection of two or more pathogens in one respiratory specimen.

### Statistical analysis

Data were analyzed using R software (version 4.3.2) and SPSS software (version 27.0). Categorical variables are presented as numbers and percentages [*n* (%)]. The chi-square test was used to compare detection rates among different regions, sexes, age groups, and seasons. Fisher’s exact test was applied when expected cell counts were less than 5. Multivariable logistic regression was performed to identify factors associated with pathogen detection. Variables included region, sex, age group, and season. Adjusted odds ratios (aORs) and 95% confidence intervals (CIs) were calculated. All tests were two-sided, and *p* < 0.05 was considered statistically significant.

## Results

### Overall detection rates

Among 5,835 respiratory samples, 3,143 were positive for at least one pathogen. The overall detection rate was 53.9%. Single-pathogen detection occurred in 2,853 samples (48.9%), and co-detection occurred in 290 samples (5.0%). The most frequently detected pathogens were RV (1,390/5,835, 23.8%), RSV (1,178/5,835, 20.2%) and Flu A (514/5,835, 8.8%). ADV, MP and Flu B were detected in 275 (4.7%), 65 (1.1%) and 22 (0.4%) samples, respectively (Table 1).

**Table 1:** Overall detection rates of respiratory pathogens in children from Xiamen and Linxia.

Variable	RV	RSV	Flu A	ADV	MP	Flu B
Number of positive cases	1,390	1,178	514	275	65	22
Detection rate (%)	23.8	20.2	8.8	4.7	1.1	0.4

Because pathogen-specific detections were not mutually exclusive, the total number of detections exceeded the number of positive samples.

### Regional differences

Overall positivity was significantly higher in Linxia than in Xiamen (65.4% vs. 49.0%, *χ*^2^ = 129.53, *p* < 0.001). The detection rates of RV, Flu A, ADV and MP also differed significantly between the two regions, whereas RSV and Flu B did not (Table 2).

**Table 2:** Detection rates of six respiratory pathogens by region [*n* (%)].

Region	Number of cases	Overall detection rate (%)	RV	RSV	Flu A	ADV	MP	Flu B
Xiamen	4,109	2015 (49.0)	900 (21.9)	811 (19.7)	238 (5.8)	148 (3.6)	35 (0.9)	18 (0.4)
Linxia	1726	1,128 (65.4)	490 (28.4)	367 (21.3)	276 (16.0)	127 (7.4)	30 (1.7)	4 (0.2)
*χ*^2^ value		129.53	27.822	1.6629	156.11	37.355	7.882	0.883
*p* value		<0.001	<0.001	0.197	<0.001	<0.001	<0.01	0.347

Pathogen-specific detections were not mutually exclusive; their sum may exceed the overall number of positive samples.

In Xiamen, 4,109 samples were tested, of which 2,015 (49.0%) were positive for at least one pathogen. RV, RSV and Flu A were the three most frequently detected pathogens. MP and Flu B had relatively low detection rates in Xiamen (Table 2).

In Linxia, 1,726 samples were tested, of which 1,128 (65.4%) were positive. RV, RSV and Flu A were again the leading pathogens. Compared with Xiamen, Linxia had higher detection rates for most pathogens, particularly Flu A and ADV (Table 2).

### Detection by sex

Overall positivity was modestly but significantly higher in males than in females (55.6% vs. 51.4%, *p* < 0.01). Among the six pathogens, RV detection was higher in males than in females (*p* < 0.01). No significant sex-related differences were observed for RSV, Flu A, ADV, MP or Flu B (*p* > 0.05) (Table 3).

**Table 3:** Detection rates of six respiratory pathogens by sex, age group and season [*n* (%)].

Variable	Number of cases	Overall detection rate (%)	RV	RSV	Flu A	ADV	MP	Flu B
Sex								
Male	3,458	55.6	870 (25.2)	701 (20.3)	317(9.2)	159 (4.6)	41 (1.2)	13 (0.4)
Female	2,377	51.4	520 (21.9)	477 (20.1)	197(8.3)	116 (4.9)	24 (1.0)	9 (0.4)
*χ*^2^ value		9.897	8.185	0.025	1.249	0.191	0.252	0.00
*p* value		<0.01	<0.01	0.874	0.264	0.662	0.615	1.000
Age group								
Infants	2,306	48.0	454 (19.7)	596 (25.8)	98 (4.2)	40 (1.7)	8 (0.3)	7 (0.3)
Toddlers	1,655	59.5	433 (26.2)	369 (22.3)	151 (9.1)	108 (6.5)	13 (0.8)	4 (0.2)
Preschool children	1,278	60.0	367 (28.7)	199 (15.6)	161 (12.6)	98 (7.7)	25 (2.0)	5 (0.4)
School-aged children	596	48.0	136 (22.8)	14 (2.3)	104 (17.4)	29 (4.9)	19 (3.2)	6 (1.0)
*χ*^2^ value		80.899	43.921	184.99	138.11	82.545	45.439	7.437
*p* value		<0.001	<0.001	<0.001	<0.001	<0.001	<0.001	0.059
Season								
Spring	1,383	41.6	384 (27.8)	147 (10.6)	18 (1.3)	75 (5.4)	14 (1)	0(0)
Summer	1,214	52.1	231 (19.0)	367 (30.2)	4 (0.3)	54 (4.4)	9 (0.7)	8 (0.7)
Autumn	1705	60.3	444 (26.0)	415 (24.3)	200 (11.7)	47 (2.8)	4 (0.2)	10 (0.6)
Winter	1,533	59.1	331 (21.6)	249 (16.2)	292 (19)	99 (6.5)	38 (2.5)	4 (0.3)
*χ*^2^ value		129.76	36.057	187.47	423.87	26.667	39.552	10.345
*p* value		<0.001	<0.001	<0.001	<0.001	<0.001	<0.001	0.016

Data are presented as *n* (%). Pathogen-specific detections were not mutually exclusive; their sum may exceed the overall number of positive samples.

### Detection by age group

Overall positivity differed significantly by age group (*χ*^2^ = 80.899, *p* < 0.001). The rates were 48.0% in infants, 59.5% in toddlers, 60.0% in preschool children and 48.0% in school-aged children. The highest overall positivity was observed in preschool children, followed by toddlers (Table 3).

Pathogen-specific patterns also differed by age. RSV had the highest detection rate in infants (25.8%), whereas RV was most frequent in preschool children (28.7%) and toddlers (26.2%). Flu A and MP increased with age and were relatively more frequent in school-aged children. Flu B showed no significant difference across age groups (*p* = 0.059) (Table 3).

### Detection by season

**Figure 1:**
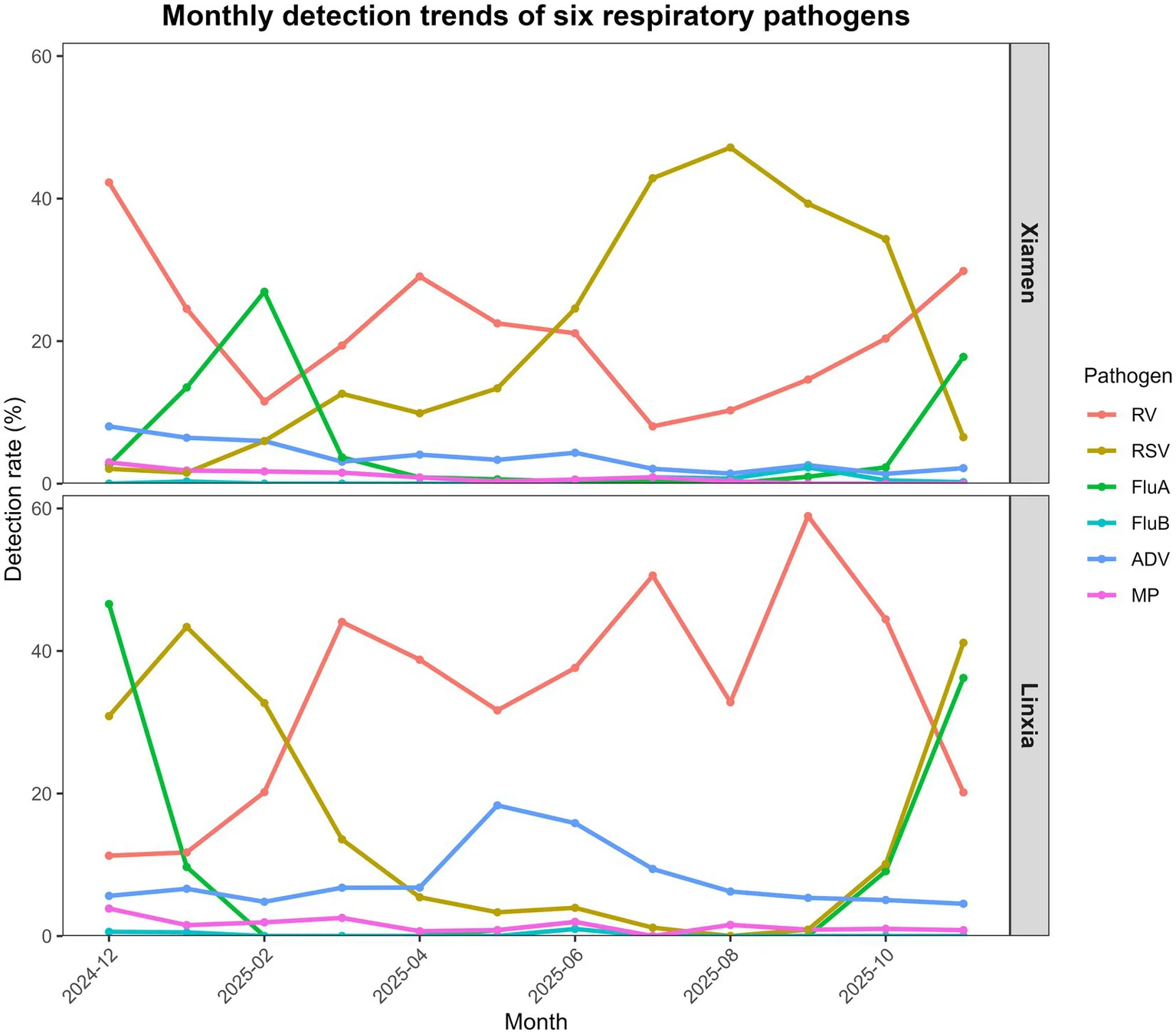
Monthly detection trends of six respiratory pathogens in Xiamen and Linxia.

Overall positivity varied significantly by season (*χ*^2^ = 129.76, *p* < 0.001). Detection was lowest in spring (41.6%) and higher in summer (52.1%), autumn (60.3%) and winter (59.1%). All six pathogens showed significant seasonal variation (Table 3, Figure 1).

RV was detected throughout the year, with higher overall rates in spring and autumn than in summer. RSV detection was highest in summer overall, whereas Flu A was most frequent in autumn and winter. Regional patterns were broadly similar for most pathogens; however, the timing of peak activity differed between the two sites for both RSV and influenza A. RSV activity was highest from summer to early autumn in Xiamen but during colder months in Linxia, while influenza A also reached peak activity in different months in the two regions. In the month-level analysis of autumn and winter (Supplementary Table S2), overall positivity in Xiamen declined across autumn (58.1, 55.1, and 52.2% from September to November) and was 55.7, 44.8, and 49.6% from December to February. RSV predominated in September and October, RV from November to January, and Flu A in February. In Linxia, overall positivity increased across autumn (60.7, 66.7, and 86.4%) and then declined across winter (81.0, 65.3, and 53.8%). RV predominated in September and October, followed by RSV in November and Flu A in December, while RSV again predominated in January and February. MP detection was uncommon in autumn (0.2% overall) but increased in winter (2.5%), with the highest monthly rates observed in December in both Xiamen (3.0%) and Linxia (3.9%).

### Multivariable analysis

After adjustment for sex, age group and season, children in Linxia had higher odds of pathogen detection than those in Xiamen (aOR 1.86, 95% CI 1.65–2.10; *p* < 0.001) (Table 4). Male sex was also associated with a higher likelihood of detection (aOR 1.21, 95% CI 1.08–1.34; *p* < 0.001). Compared with infants, toddlers (aOR 1.50, 95% CI 1.31–1.70; *p* < 0.001) and preschool children (aOR 1.47, 95% CI 1.28–1.70; *p* < 0.001) had higher odds of pathogen detection, whereas school-aged children did not differ significantly from infants (aOR 0.90, 95% CI 0.75–1.09; *p* = 0.283). Compared with spring, detection odds were higher in summer (aOR 1.62, 95% CI 1.38–1.90; *p* < 0.001), autumn (aOR 2.19, 95% CI 1.89–2.53; *p* < 0.001) and winter (aOR 1.90, 95% CI 1.64–2.21; *p* < 0.001).

**Table 4:** Multivariable logistic regression analysis of factors associated with respiratory pathogen detection.

Variable	aOR	95% CI	*p* value
Region			
Linxia vs. Xiamen	1.86	1.65–2.10	<0.001
Sex			
Male vs. Female	1.21	1.08–1.34	<0.001
Age group			
Toddler vs. Infant	1.50	1.31–1.70	<0.001
Preschool vs. Infant	1.47	1.28–1.70	<0.001
School-aged vs. Infant	0.90	0.75–1.09	0.283
Season			
Summer vs. Spring	1.62	1.38–1.90	<0.001
Autumn vs. Spring	2.19	1.89–2.53	<0.001
Winter vs. Spring	1.90	1.64–2.21	<0.001

aOR, adjusted odds ratio; CI, confidence interval.

Reference groups: Xiamen (region), Female (sex), Infant (age group), and Spring (season).

## Discussion

This multicenter study found that more than half of children with ARIs tested positive for at least one of the six respiratory pathogen targets examined. RV, RSV and Flu A were the most frequently detected targets in both Xiamen and Linxia. Overall, positivity was higher in inland Linxia than in coastal Xiamen, and this association persisted after adjustment for sex, age group and season. The observed difference may reflect a combination of environmental conditions, population density, urbanization, socioeconomic characteristics, healthcare access and local testing practices (15, 16). The relatively high detection rates observed in this study may also reflect the resurgence of respiratory viral circulation following the relaxation of COVID-19 non-pharmaceutical interventions, as reported in previous studies (15, 17, 18).

The overall and pathogen-specific detection rates varied in different studies. A multi-center data on common viral respiratory infections in south China showed that at least one virus was detected in 1099 (24.96%) samples by direct immunofluorescence antibody assay. The detection rates were 7.13% (314/4403) and 5.31% (234/4403) for RSV and IFA (19). A pediatric study in tropical Panama identified RSV, influenza A and RV as the leading viruses. RSV was detected mainly from July to November and influenza from May to July (7). In Egypt, a multicenter study of hospitalized children with severe acute respiratory infections reported 72.7% positivity using a 33-pathogen panel, with RSV accounting for 63.8% of virus-positive cases (5). In Nepal, an expanded molecular panel detected at least one pathogen in 98.7% of young children hospitalized with severe pneumonia; RV and RSV were predominant. RSV outbreaks occurring mainly at the end of the monsoon or during winter (20). Together, these studies support the recurrent importance of RV, RSV and influenza in pediatric respiratory disease while demonstrating that absolute detection rates and epidemic timing are highly context-dependent. The absolute detection rates are not directly comparable because of differences in age distribution, disease severity, clinical setting, specimen type, diagnostic method, panel breadth and study period.

Male sex was associated with a modest increase in overall detection, and RV was the only pathogen with a significant sex-related difference in the pathogen-specific analyses. Our finding of higher positivity among male children are consistent with previously published studies on respiratory infections (21, 22) Nevertheless, this observation cannot be simply attributed to intrinsic biological predisposition to viral infection in males. Potential contributing factors may include sex-based differences in immune responses and other unmeasured variables (22, 23). The underlying mechanisms remain unclear and warrant further prospective evaluation.

The age-related findings are consistent with differences in exposure and immunity across childhood. The increased risk observed in toddlers and preschool children is consistent with their greater exposure in daycare and kindergarten settings. Close contact, immature hygiene behaviors, and increased social interaction likely contribute to higher transmission in these age groups. In contrast, no significant difference was observed in school-aged children after adjustment, suggesting that accumulated immunity and behavioral factors may reduce susceptibility in older children.

Seasonal variation was pronounced and remained significant in multivariable analysis. Compared with spring, detection risk was highest in autumn (aOR = 2.19), followed by winter and summer. Seasonal changes in temperature, humidity, school attendance and indoor crowding may contribute to these patterns. The persistence of seasonal effects after adjustment highlights the strong influence of environmental factors on pathogen transmission (24–26).

Different pathogen targets also demonstrated distinct detection patterns between the two regions. RV was the most frequently detected target in both regions, whereas RSV and influenza A virus showed more pronounced seasonal variation. These observations are broadly consistent with previous pediatric respiratory surveillance studies conducted in China and other Asian regions (16, 25, 26). Importantly, the regional difference persisted after controlling for season, suggesting that climate alone does not fully explain the observed heterogeneity. Other unmeasured environmental and population-related factors may also contribute (16, 24). Xiamen and Linxia differ simultaneously in latitude, altitude, climate, land area, population density, urbanization, income and healthcare access. The labels ‘coastal’ and ‘inland’ therefore summarize bundles of contextual characteristics rather than an isolated causal exposure. Greater population density and urbanization in Xiamen may increase the frequency and connectivity of interpersonal contacts. Conversely, Linxia’s higher altitude, colder and relatively drier winters, more dispersed population and different urban–rural structure may produce different exposure and transmission patterns. Nevertheless, overall positivity was higher in the more sparsely populated Linxia, demonstrating that population density alone cannot explain the regional difference. Moreover, the study denominator comprised children tested at the participating hospitals rather than the entire population of either region, and the hospitals’ effective catchment populations were unknown. In particular, the comparison cannot separate coastality from the north–south climatic gradient or from healthcare and sampling differences. Multi-year studies incorporating contemporaneous meteorological measurements, population-mobility indicators and harmonized sampling across multiple hospitals will be required to identify the factors responsible for the different pathogen trajectories.

Importantly, a positive upper-airway nucleic acid test identifies pathogen genetic material but does not by itself establish that the detected organism caused the concurrent ARI. We could not distinguish acute causal infection from incidental carriage, an incubation-phase detection or prolonged shedding. We therefore interpret the results as detection frequencies among children with clinically diagnosed ARIs rather than pathogen-attributable fractions.

This study has several limitations. First, its retrospective, clinician-directed convenience sample was susceptible to selection bias. The databases did not include children with ARIs who were not tested, and differences in healthcare-seeking behavior or indications for testing may have influenced both the study population and positivity estimates. Clinical information was incomplete or inconsistently recorded for some patients, particularly vaccination history, comorbidities, disease severity, clinical outcomes and healthcare utilization. These missing data limited adjustment for potential confounders and prevented a comprehensive assessment of the clinical consequences of pathogen detection. Second, only two hospitals and administrative regions were included, limiting generalizability to other coastal or inland areas of China. Center-specific differences in patient referral patterns, indications for testing, timing of specimen collection, specimen handling and laboratory workflows cannot be excluded. Third, the panel was restricted to six pathogens and did not assess SARS-CoV-2, parainfluenza virus, human metapneumovirus or most bacterial pathogens; consequently, total pathogen burden and co-detection patterns were underestimated and that included targets may have been detected alongside unmeasured pathogens. Fourth, the six investigated pathogens comprise multiple distinct subtypes or lineages. Our assay did not enable further sub-typing or lineage discrimination. Potential regional or age-related differences at the subtype level should be explored in future study. Fifth, qualitative nucleic acid detection in an upper-airway specimen cannot establish etiologic attribution or disease severity, particularly for RV and other organisms that may be detected asymptomatically or after prolonged shedding. Finally, meteorological variables, vaccination status, comorbidities, clinical outcomes and healthcare utilization patterns were not available for adjustment.

Despite these limitations, the study provides a direct comparison of pediatric respiratory pathogen detection in two geographically distinct Chinese regions during a post-pandemic-era surveillance period. The findings support the value of regional surveillance and suggest that prevention planning should consider local epidemiological patterns rather than relying only on national averages. Multi-year studies incorporating clinical severity, vaccination status, and a broader pathogen panel are warranted.

In conclusion, RV, RSV and Flu A were the most frequently detected targets among children with ARIs in both Xiamen and Linxia. Overall positivity for the six-target panel was higher in Linxia than in Xiamen. Age group and season were independently associated with detection. These findings highlight regional, age-related and seasonal heterogeneity in pediatric respiratory pathogen detection and support locally tailored surveillance and prevention strategies.

## Data Availability

The raw data supporting the conclusions of this article will be made available by the authors, without undue reservation.
