# Supplementary material for: Comparative epidemiology of six respiratory pathogens among children aged 28 days to 14 years with acute respiratory infections in coastal Xiamen and inland Linxia, China: a multicenter retrospective study
Source: Front Public Health. 2026 Sep 3;14:1926943. doi: 10.3389/fpubh.2026.1926943 (PMC13582421; doi:10.3389/fpubh.2026.1926943)
Supplement: Supplementary file 1 [file Data_Sheet_1.docx]

**Supplementary Table S1 Contextual characteristics of the two study settings**

| **Indicator** | **Xiamen** | **Linxia** |
| --- | --- | --- |
| Geographic setting | Southeastern coastal city;  subtropical maritime monsoon climate | Northwestern inland plateau prefecture;  cold temperate semi-arid climate or cold temperate semi-humid climate |
| Administrative land area | 1,700.61 km^2^ | 8,169 km^2^ |
| Altitude | Coastal, predominantly low-altitude | Mean approximately 2,000 m |
| Permanent population, 2025 | 5.365 million | 2.072 million |
| Urbanization rate, 2025 | 91.31% | 43.09% |
| Annual GDP, 2025 | CNY 898.037 billion | CNY 54.66 billion |
| Per-capita disposable income, 2025 | CNY 77,762 | CNY 18,566 |
| Medical and health institutions, 2025 | 2,786 | 1,813 |
| Medical beds, 2025 | 25,220 | 17,757 |

**Supplementary Table S2 Monthly detection of six respiratory pathogens among children with acute respiratory infections during autumn and winter in Xiamen and Linxia**

| Region | Season | Month | Samples | Any pathogen detected | RV | RSV | Flu A | ADV | MP | Flu B |
| --- | --- | --- | --- | --- | --- | --- | --- | --- | --- | --- |
| Xiamen | Autumn | Sep | 308 | 179 (58.1%) | 45 (14.6%) | 121 (39.3%) | 3 (1.0%) | 8 (2.6%) | 0 (0.0%) | 7 (2.3%) |
| Xiamen | Autumn | Oct | 437 | 241 (55.1%) | 89 (20.4%) | 150 (34.3%) | 10 (2.3%) | 6 (1.4%) | 0 (0.0%) | 2 (0.5%) |
| Xiamen | Autumn | Nov | 506 | 264 (52.2%) | 151 (29.8%) | 33 (6.5%) | 90 (17.8%) | 11 (2.2%) | 0 (0.0%) | 1 (0.2%) |
| Xiamen | Winter | Dec | 336 | 187 (55.7%) | 142 (42.3%) | 7 (2.1%) | 9 (2.7%) | 27 (8.0%) | 10 (3.0%) | 0 (0.0%) |
| Xiamen | Winter | Jan | 326 | 146 (44.8%) | 80 (24.5%) | 5 (1.5%) | 44 (13.5%) | 21 (6.4%) | 6 (1.8%) | 1 (0.3%) |
| Xiamen | Winter | Feb | 234 | 116 (49.6%) | 27 (11.5%) | 14 (6.0%) | 63 (26.9%) | 14 (6.0%) | 4 (1.7%) | 0 (0.0%) |
| Linxia | Autumn | Sep | 112 | 68 (60.7%) | 66 (58.9%) | 1 (0.9%) | 0 (0.0%) | 6 (5.4%) | 1 (0.9%) | 0 (0.0%) |
| Linxia | Autumn | Oct | 99 | 66 (66.7%) | 44 (44.4%) | 10 (10.1%) | 9 (9.1%) | 5 (5.1%) | 1 (1.0%) | 0 (0.0%) |
| Linxia | Autumn | Nov | 243 | 210 (86.4%) | 49 (20.2%) | 100 (41.2%) | 88 (36.2%) | 11 (4.5%) | 2 (0.8%) | 0 (0.0%) |
| Linxia | Winter | Dec | 337 | 273 (81.0%) | 38 (11.3%) | 104 (30.9%) | 157 (46.6%) | 19 (5.6%) | 13 (3.9%) | 2 (0.6%) |
| Linxia | Winter | Jan | 196 | 128 (65.3%) | 23 (11.7%) | 85 (43.4%) | 19 (9.7%) | 13 (6.6%) | 3 (1.5%) | 1 (0.5%) |
| Linxia | Winter | Feb | 104 | 56 (53.8%) | 21 (20.2%) | 34 (32.7%) | 0 (0.0%) | 5 (4.8%) | 2 (1.9%) | 0 (0.0%) |

Note: Data are presented as n (%), with percentages calculated using the total number of samples tested in each region and month as the denominator. “Any pathogen detected” indicates detection of at least one of the six tested pathogens. Pathogen-specific detections were counted independently; therefore, their percentages may not sum to the percentage of samples with any pathogen detected. Co-detections were counted separately for each pathogen.
